# Well-Differentiated Extraskeletal Osteosarcoma Arising from the Retroperitoneum That Recurred as Anaplastic Spindle Cell Sarcoma

**DOI:** 10.1155/2010/327591

**Published:** 2010-03-04

**Authors:** Hiromasa Arai, Yasushi Rino, Teppei Nishii, Norio Yukawa, Nobuyuki Wada, Hisashi Oshiro, Tsuyoshi Ishida, Noboru Nakaigawa, Munetaka Masuda

**Affiliations:** ^1^Department of Pulmonary Medicine (Surgery), Yokohama City University Medical Center, 4-57 Urafune-cho, Minami-ku, Yokohama, 232-0024, Japan; ^2^Department of Surgery, Yokohama City University School of Medicine, 3-9 Fukuura, Kanazawa-ku, Yokohama, 236-0004, Japan; ^3^Department of Pathology, Yokohama City University School of Medicine, 3-9 Fukuura, Kanazawa-ku, Yokohama, 236-0004, Japan; ^4^Department of Pathology and Laboratory Medicine, Kohnodai Hospital, National Medical Center of Japan, 1-7-1 Kohnodai, Ichikawa, Chiba, 272-8516, Japan; ^5^Department of Urology, Yokohama City University School of Medicine, 3-9 Fukuura, Kanazawa-ku, Yokohama, 236-0004, Japan

## Abstract

Extraskeletal osteosarcoma is an uncommon high-grade malignant soft tissue sarcoma. Well-differentiated extraskeletal osteosarcoma is thought to have a better prognosis than classical extraskeletal osteosarcoma, but dedifferentiation after recurrence has also been reported. We present a case of a primary retroperitoneal extraskeletal osteosarcoma in a 62-year-old Japanese woman. Abdominal CT revealed a large mass with diffuse calcification in the right retroperitoneal space and tumor resection was performed. The histopathological diagnosis was well-differentiated retroperitoneal extraskeletal osteosarcoma. She was followed up by CT every 6 months without adjuvant radiotherapy and chemotherapy for 31 months until anaplastic high-grade spindle cell sarcoma recurred in the retroperitoneum. Our case is the seventh reported description of well-differentiated extraskeletal sarcoma, and the first to arise in the retroperitoneum and recur as an entirely dedifferentiated spindle cell sarcoma.

## 1. Introduction

Well-differentiated extraskeletal osteosarcoma is a rare soft tissue sarcoma, with only 6 cases reported in the English literature [1–4]. The biological behavior of this tumor has been suggested to be better than that of classical extraskeletal osteosarcoma, but some cases can progress to a higher grade and may result in death [4]. Only 2 cases of dedifferentiation of the neoplasm as a recurrent tumor have been reported. Here, we report a case of well-differentiated extraskeletal osteosarcoma arising from the retroperitoneum, which recurred in the retroperitoneum as an entirely dedifferentiated spindle cell sarcoma 31 months after the initial surgery.

## 2. Case Presentation

**Figure 1:**
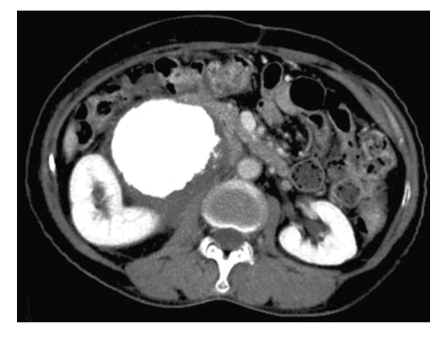
Abdominal CT revealed a large mass with diffuse calcification in the right retroperitoneal space.

A 62-year-old woman was admitted to our hospital complaining of severe right abdominal pain for 4 months. A fixed stony-hard tumor of about 100 mm in diameter was found on palpation, with slight tenderness on the right side of her abdomen. A full blood examination gave values within the normal limit. Abdominal CT showed a large mass with diffuse calcification of size about 130 × 110 mm in the right retroperitoneal space between the right kidney and the inferior vena cava, with slight hydronephrosis (Figure 1). Lymph node swelling, ascites, liver tumor, and lung tumor were not detected by chest and abdominal CT, and the patient had no history of trauma or radiation therapy at the tumor site. However, the patient had undergone bilateral resection of tumors of the adrenal glands on the left side at the age of 46 years old and on the right side at at the age of 50 years old. Pathological findings revealed that both adrenal gland tumors were pheochromocytoma and no adjuvant therapy was administered. This led to a preoperative diagnosis of recurrent pheochromocytoma or retroperitoneal osteosarcoma or leiomyosarcoma. There were no carcinomas or sarcomas in her history or in her family history. 

Complete surgical resection of the primary tumor was performed. The tumor was situated behind the mesenterium of the ascending colon, compressing the right kidney, but no direct invasion of the surrounding organs was observed: there was no lymph node swelling, ascites or peritoneal dissemination, and the tumor was not present in the parenchyma of the liver and the kidney. The surgical margin was determined based on the rough connective tissue surrounding the tumor. Dissection of lymph nodes was not performed.

**Figure 2:**
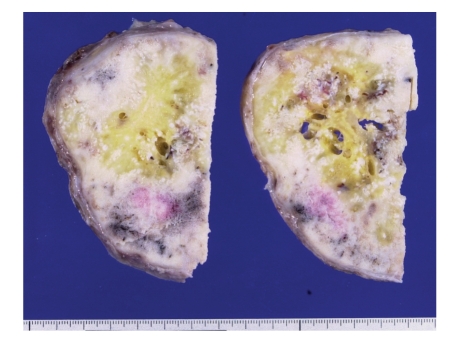
The cut surface of the retroperitoneal tumor, showing a solid, bony, and yellowish-white mass.

**Figure 3:**
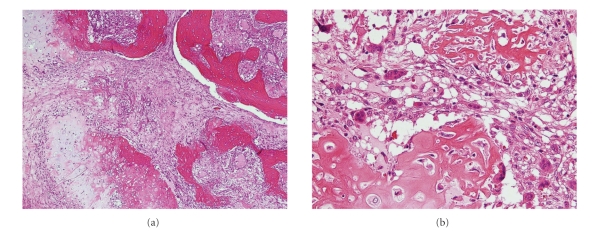
(a) Low-power photomicrograph of the primary tumor, showing irregularly arranged woven bone trabeculae and atypical cartilage islands along with intervening fibrous tissue. (b) High-power photomicrograph, showing irregular osteoid seams with atypical osteoblasts and atypical chondrocytes within the cartilage matrix. These findings are consistent with low-grade osteosarcoma.

Gross examination revealed a rounded, bony, and hard tumor of size about 140 × 110 × 80 mm and weight 1100 g that was yellowish-white in color (Figure 2). Histologically, the tumor consisted of a mixture of dense fibrous tissue, woven bone, and atypical cartilage. Some osteoclastic giant cells were observed. Woven bone trabeculae were arranged irregularly and were anastomosed to each other. The architecture of the woven bone trabeculae displayed a reverse zoning phenomenon, with mature bony tissue in the center and less mature bony tissue in the periphery. Although the cellularity of spindle cells within the fibrous tissue was not greatly increased, these cells showed mild but definite nuclear atypism with hyperchromasia and scattered mitotic figures. There was no evidence of fatty differentiation of these cells, indicating the absence of a well-differentiated or dedifferentiated liposarcoma. Operative findings showed no continuity with skeletal bones, leading to a final diagnosis of well-differentiated (low-grade) extraskeletal osteosarcoma of the retroperitoneum (Figures 3(a) and 3(b)). 

The postoperative course was uneventful. We recommended adjuvant therapy because of the probable poor prognosis, but the patient refused radiotherapy and chemotherapy. The patient was followed up by CT every 6 months without receiving adjuvant radiation therapy or chemotherapy.

**Figure 4:**
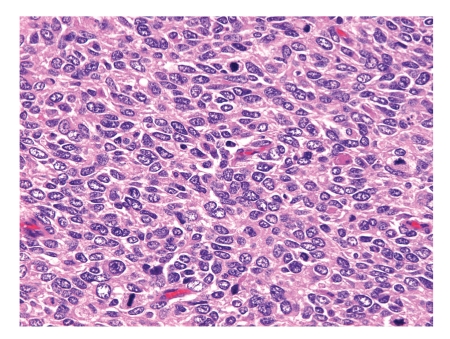
High-power photomicrograph of the recurrent tumor, showing cellular proliferation of anaplastic tumor cells with many mitotic figures.

Thirty-one months after the operation, she was readmitted to our hospital due to severe pain in the lumbar region and the right lower leg. Abdominal CT revealed a large mass in the right retroperitoneal space. No calcification was seen, but recurrence of the primary tumor was strongly suspected. Surgical resection of the recurrent tumor was performed via a transabdominal approach, in which a tumor with adherence to the right kidney was completely removed, a procedure of tumor resection and right nephrectomy. Grossly, the recurrent retroperitoneal tumor adherent to the right kidney was of size 310 × 300 mm, with necrosis and hemorrhage and a yellowish-white color on the cut surface. The recurrent tumor was characterized histologically by proliferation of atypical nonepithelial cells with hyperchromatic nuclei and a high nuclear-cytoplasmic ratio. The mitotic count of the neoplastic cells was about 50 per 10 high-power fields. Anaplastic features predominated, but a few foci of matrix production looked like osteoid or hyalinized collagen within the recurrent neoplastic tissues. These findings led to a diagnosis of high-grade malignant anaplastic spindle cell sarcoma, consistent with the recurrence of extraskeletal osteosarcoma (Figure 4). There were no malignant findings in the kidney. The abdominal CT scan did not show an apparent recurrent tumor until the 30th month after the initial operation and there were no established tumor markers for extraskeletal osteosarcoma. We thought that the recurrent tumor had increased rapidly in one month and the pathological findings for the tumor were compatible with this conclusion. The patient was discharged on the 18th postoperative day, but 2 months later she was re-admitted due to severe back pain. Oxycodone hydrochloride was administered orally, but gradually she complained of dyspnea and received intravenous morphine hydrochloride for sedation. Chest and abdominal CT showed a lesion corresponding to the location of the primary tumor, together with multiple lung nodules and right pleural effusion. Gradually her general status worsened and the patient died due to widespread metastasis 33 months after the initial operation.

## 3. Discussion

Extraskeletal osteosarcoma is defined as a malignant osseous neoplasm arising from soft tissue without attachment to skeletal bones. This neoplasm is rare compared with bone osteosarcoma, accounting for 1-2% of all soft tissue sarcomas [5], and has a calculated annual incidence of 2 to 3 per million population [5]. Diagnosis rests on three criteria: (1) the presence of a uniform morphological pattern of sarcomatous tissues that excludes the possibility of mixed malignant mesenchymal tumor, (2) production by sarcomatous tissues of malignant osteoid or bone (or both), and (3) ready exclusion of an osseous origin [6]. Extraskeletal osteosarcoma occurs in older adults compared with skeletal osteosarcoma (median ages of 55 and 20 years old, respectively) [5] and may have an intramuscular or a superficial location, with the thigh being the most common site (42.3%–52%) [5, 7], followed by the upper extremities (11.5%) [7], the retroperitoneum (11.5%) [7], and the buttocks (7.7%) [7]. The duration of the symptoms (enlargement of soft tissue mass, pain) varies from several weeks to several years [5, 7–9]. The pathogenesis of the tumor is unclear, but radiation-induced extraskeletal osteosarcoma [5, 7, 10, 11], a history of preceding trauma at the tumor site [5, 7, 11], and malignant transformation of myositis ossificans to extraskeletal osteosarcoma [7, 11] have been proposed.

Extraskeletal osteosarcoma is generally a highly aggressive neoplasm with a grave prognosis, and its histology is basically high-grade, similar to most skeletal osteosarcomas. Most patients die from metastatic disease within 2 to 3 years of the initial diagnosis [5, 8, 9], with a reported local recurrence rate of 45%–50% and distant metastasis in over 60% of cases [7, 12]. Metastasis within two years after surgery has been found in 80% of the cases, with a five-year survival rate of 13% to 37% [9]. Bane et al. concluded that tumor size (<5 versus ≥5 cm) is the major predictor of survival [7].

It is not always easy to distinguish extraskeletal osteosarcoma from other benign or malignant bone- and cartilage-forming soft tissue lesions [5]. Differential diagnosis requires consideration of various soft tissue osteogenic lesions [2], including myositis ossificans, heterotopic and metaplastic ossification, pseudosarcomatous proliferative lesions, dermatomyositis with ossification, ossifying fibromyxoid tumor of soft tissue, synovial sarcoma with calcification, and malignant fibrous histiocytoma with osteoid production. Among malignant tumors, metaplastic bone is often observed in synovial sarcoma, epithelioid sarcoma, malignant fibrous histiocytoma, liposarcoma, malignant melanoma, and other mesenchymal or epithelial neoplasms [5]. The main histological differential diagnoses are soft tissue chondroma, chondrosarcoma in soft tissue, and myositis ossificans [3]. Pathological findings of a “reverse zoning phenomenon” (central deposition of osteoid material and atypical spindle cell proliferation at the periphery) [5] is especially common in sarcoma, while a “zoning phenomenon” is a characteristic of myositis ossificans [3, 5].

Treatment for extraskeletal osteosarcoma is usually restricted to local intervention, including surgery alone or a combination of surgery and radiation therapy. Systemic therapy including adjuvant chemotherapy has not been established. Patel et al. [13] summarized the efficiency of neo- and adjuvant chemotherapy consisting of doxorubicin, cisplatin, ifosfamide, methotrexate, and etoposide in 12 cases, of which only 3 showed a minor response and 9 showed no change or progression. Thus, classical, high-grade extraskeletal osteosarcoma is a rare and very aggressive tumor with a poor prognosis.

Well-differentiated (low-grade) extraskeletal osteosarcoma is extremely rare. This tumor was first described in 1953 by Umiker [14] and appears to represent a low-grade variant of osteosarcoma with better biological behavior than classical extraskeletal osteosarcoma [4]. A PubMed search found only 6 cases of well-differentiated extraskeletal osteosarcoma in the English literature. Including our case, the clinical features of the 7 reported cases of this tumor are summarized in Table 1. The patients were 2 males (28.6%) and 5 females (71.4%), and ranged in age from 32 to 74 years old (mean age 49.1 years old, median age 53 years old). This suggests a tendency of more frequent occurrence in females, with a similar onset age to classical extraskeletal osteosarcoma (median age 53 versus 55 years old). Three cases (cases 1, 6, and the present case) recurred as high-grade malignancy. All cases had a poor prognosis, with widespread metastasis after the initial surgery or death. To our knowledge, our case is the first example of a well-differentiated extraskeletal osteosarcoma arising from the retroperitoneum that recurred as anaplastic sarcoma.

Well-differentiated extraskeletal sarcoma may have a better prognosis than classical extraskeletal sarcoma, but some cases may recur as a high-grade malignancy with rapid progression leading to death. In our case, regular Follow-up was performed only with a plain abdominal CT scan at 6-month intervals. Based on our experience, we recommend closer follow-up using a chest-abdominal CT scan, abdominal magnetic resonance imaging (MRI), or ultrasound enhanced with a contrast agent.

Our case suggests that careful observation for the recurrence of the tumor is necessary if the pathological diagnosis is well-differentiated extraskeletal osteosarcoma.

## Figures and Tables

**Table 1:** Summary of reported cases with well-differentiated extraskeletal osteosarcomas.

Case	Year	First author	Age	Sex	Site	Size (cm)	Treatment	Follow-up and outcome
1	1953^a^	Umiker	44	M	Rt. thigh	5	resection	Died with widespread metastasis after 5 years
2	1989	Present	57	F	Lt. knee	24	resection	Alive 5 years after surgery
3	1991	Yi	74	F	Lt. axilla	15	resection	Alive 2 years after surgery
4	2003	Okada	35	F	Lt. leg	11	resection	Alive 4 years after surgery
5	2005	Abramovici	40	F	Lt. upper back	9 × 6 × 5	resection	Alive 2 months after surgery lost in follow-up
6	2005^a^	Abramovici	32	M	Bil. thighs, buttocks and lt. paravertebral area (max. lesion)	16 × 7 × 8	resection and chemo	Alive with widespread metastasis 4 years after initial surgery
7	Present case^a^		62	F	Retroperitoneum	14 × 11 × 8	resection	Died with widespread metastasis after 33 months

chemo: chemotherapy

^a^: recurred as a high-grade malignancy
